# Updated evidence on epidemiology, diagnosis, and treatment for colonic diverticular bleeding

**DOI:** 10.1002/deo2.70122

**Published:** 2025-05-06

**Authors:** Chikamasa Ichita, Takaaki Kishino, Tomonori Aoki, Tomohiko Machida, Takashi Murakami, Yoshinori Sato, Naoyoshi Nagata

**Affiliations:** ^1^ Gastroenterology Medicine Center Shonan Kamakura General Hospital Kanagawa Japan; ^2^ Department of Health Data Science Yokohama City University Kanagawa Japan; ^3^ Department of Gastroenterology and Hepatology Center for Digestive and Liver Diseases Nara City Hospital Nara Japan; ^4^ Department of Gastroenterology Graduate School of Medicine The University of Tokyo Tokyo Japan; ^5^ Department of Surgery Saiseikai Hyogo Prefectural Hospital Hyogo Japan; ^6^ Department of Gastroenterology Juntendo University School of Medicine Tokyo Japan; ^7^ Division of Gastroenterology St Marianna University School of Medicine Kanagawa Japan; ^8^ Department of Gastroenterological Endoscopy Tokyo Medical University Tokyo Japan

**Keywords:** colonic diverticular bleeding, colonic diverticular hemorrhage, endoscopic hemostasis, epidemiology, lower gastrointestinal bleeding

## Abstract

Since 2020, multiple large‐scale studies (CODE BLUE‐J) in Japan have accelerated the accumulation of evidence on colonic diverticular bleeding (CDB). This review summarizes the latest findings regarding CDB epidemiology and endoscopic hemostasis. Recent data show that CDB has become the most common cause of lower gastrointestinal bleeding in Japan, driven by an aging population and the increased use of antithrombotic medications. Although 70%–90% of patients achieve spontaneous hemostasis, rebleeding occurs in up to 35% of cases within 1 year. Despite an overall mortality rate of < 1%, patients with CDB can present with hypovolemic shock and may require urgent intervention. There are no effective pharmacological treatments for controlling CDB. Therefore, endoscopic therapy plays a crucial role in its management. Based on available evidence, both clipping and endoscopic band ligation are considered effective initial treatments. Recent studies indicate that direct clipping reduces early rebleeding compared with indirect clipping, while endoscopic band ligation achieves lower rebleeding rates (13%–15%) than clipping. The choice between direct clipping and endoscopic band ligation depends on the diverticulum location and the presence of active bleeding. Newer techniques, such as over‐the‐scope clip and self‐assembling peptide application, have shown potential, but require further study. The detection of the bleeding source remains challenging because accurate identification is essential for successful hemostasis. Additional research is needed to refine the endoscopic diagnostic and therapeutic techniques, prevent rebleeding, and improve patient outcomes.

## INTRODUCTION

Colonic diverticular bleeding (CDB) is the most common cause of lower gastrointestinal bleeding (LGIB), accounting for over 60% of hematochezia cases.^1^ Although CDB typically presents as transient bleeding with spontaneous resolution, a substantial proportion of patients experience recurrent bleeding. Compared to other LGIB conditions, CDB is more likely to result in severe clinical presentations such as hypovolemic shock.^2^, 

Unlike upper gastrointestinal bleeding, which has well‐established preventative strategies such as *Helicobacter pylori* eradication and proton pump inhibitors (PPIs), LGIB lacks standardized preventive measures. Endoscopic hemostasis using various techniques is critical for managing CDB. A comprehensive review conducted by Kaise et al., in 2020, provided an overview of these methods.^3^ Moreover, several large‐scale and multicenter collaborative research studies were published, updated, and expanded in this field.

This review aims to provide an updated overview of the latest epidemiology of CDB in Japan and advancements in endoscopic hemostasis.

## RECENT EPIDEMIOLOGY OF CDB IN JAPAN

### Is CDB increasing?

Previous epidemiological studies on CDB incidence trends in Japan are limited. Indirect indicators from cohort studies suggest an increasing prevalence: the proportion of CDB among patients with diverticulosis increased from 1% (22/2157) in 2003 to 1.7% (69/4159) in 2011,^4^ and CDB representation in LGIB increased from 5.9% (49/828) in 1995–2006 to 23% (224/975) in 2007–2013.^5^

**FIGURE 1:**
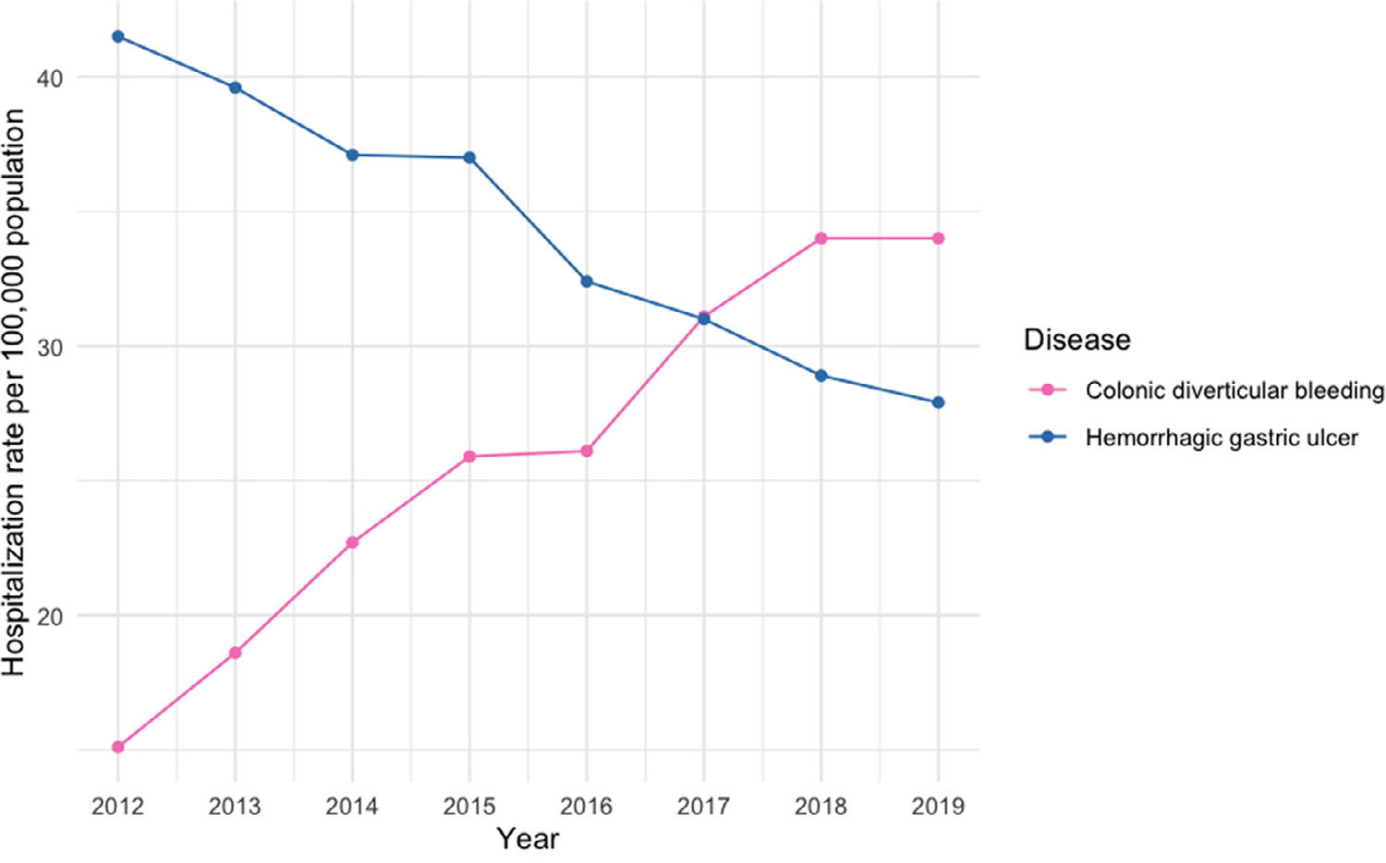
National trends in hospitalizations for colonic diverticular bleeding and hemorrhagic gastric ulcers in Japan from 2012 to 2019^†^. ^†^Partially modified from Figure 1 in Reference 6.

A research study using the National Database, which comprehensively covers the Japanese insured population, revealed a dramatic increase in CDB hospitalizations, which exhibited more than doubled values from 15.1 per 100,000 individuals in 2012 to 34.0 per 100,000 in 2019 (Figure 1).^6^ Concurrently, hospitalizations for hemorrhagic gastric ulcers declined significantly, decreasing from 41.5 per 100,000 in 2012 to 27.9 per 100,000 in 2019, with a notable shift observed in 2017.^6^ This epidemiological transition can be attributed to divergent preventive capabilities. While upper gastrointestinal bleeding can be mitigated through *H. pylori* eradication andproton pump inhibitors, CDB risk appears to be escalating due to population aging and increased antithrombotic medication use.

### Age distribution of CDB

Age distribution data for CDB in Japan are scarce. A single‐center study from 2003 to 2011 reported the age distribution of CDB as follows: under 39 years, 0.1%; 40–59 years, 19.2%; and ≥60 years, 80.6%.^4^ In contrast, the National Database analysis of CDB hospitalizations showed a different distribution: 17% under 65 years, 39.5% for 65–79 years, and 43.5% aged 80 and above, with a peak in the 80–84 age group.^6^

### Mortality of CDB

Three large‐scale studies in Japan consistently showed that the mortality rate associated with CDB is approximately 0.5%. A study using Diagnosis Procedure Combination (DPC) data from 2010–2012 investigating LGIB found an overall in‐hospital mortality of 2.5% (782/30,846), with CDB‐specific mortality at 0.7% (62/8422).^7^ This research demonstrated that mortality rates for CDB were higher among elderly males, but lower compared to other LGIB causes. Another investigation using DPC data from 2010 to 2017 showed an in‐hospital mortality of 0.45% (249/55,392 cases) for CDB.^8^ A nationwide multicenter study further reported an in‐hospital mortality of 0.2% (16/6575).^1^ These findings collectively indicate that CDB mortality in Japan is less than 1%, suggesting it is a condition with relatively low mortality.

### Spontaneous hemostasis in CDB

CDB often resolves spontaneously after conservative management. Japanese reports, including a nationwide multicenter study, have demonstrated that 70%–90% of patients with CDB achieve spontaneous hemostasis during hospitalization.^9^, ^10^, ^11^ A single‐center retrospective study further revealed that patients with specific characteristics, such as systolic blood pressure above 90 mmHg and without extravasation on contrast computed tomography, showed an exceptionally high spontaneous hemostasis rate of 92.7% (127/137).^12^

### Short‐term and long‐term rebleeding Rate in CDB

A nationwide multicenter study reported a 30‐day rebleeding rate of 22.8% (1501/6575) for CDB.^11^ Analysis of DPC data from 2010 to 2017 indicated that 14.3% (7930/55,389) of patients required invasive procedures including interventional radiology (IVR) and surgery, in addition to colonoscopy during hospitalization due to rebleeding.^8^

Regarding long‐term rebleeding, four cohort studies, including a nationwide multicenter study, consistently reported a 1‐year rebleeding rate of 19%–35% among patients who were initially hemostatic and discharged.^10^, ^13^, ^14^, ^15^

### Summary of epidemiology of CDB in Japan

The incidence of CDB is rapidly increasing in Japan, with marked prevalence in the elderly population. Despite high spontaneous hemostasis rates and low mortality, CDB is characterized by a significant rebleeding potential. Unlike upper gastrointestinal bleeding, CDB lacks an established preventive strategy. Consequently, minimally invasive endoscopic management is crucial to prevent rebleeding, necessitating expertise in evidence‐based treatment approaches.

## ADVANCES IN THE ENDOSCOPIC MANAGEMENT OF CDB

### Is early colonoscopy appropriate for CDB?

Conduction of randomized controlled trials (RCTs) that exclusively focus on CDB is challenging, as a definitive CDB diagnosis requires colonoscopy. Therefore, the evidence discussed here is based on studies on acute LGIB. Before 2017, only four meta‐analyses, two RCTs, and five observational studies had evaluated the appropriate timing of colonoscopy for LGIB.^16^, ^17^, ^18^, ^19^, ^20^, ^21^, ^22^, ^23^, ^24^, ^25^ The Japan Gastroenterological Association guidelines for CDB and colonic diverticulitis, published in 2017, recommended performing early endoscopy within 24 h of arrival.^26^ However, by December 2024, research evaluating colonoscopy timing had expanded, with four additional meta‐analyses, two additional RCTs, and nine additional observational studies published.^8^, ^27^, ^28^, ^29^, ^30^, ^31^, ^32^, ^33^, ^34^, ^35^, ^36^, ^37^, ^38^, ^39^, ^40^ Consequently, routine early colonoscopy within 24 h is no longer recommended for LGIB.

**FIGURE 2:**
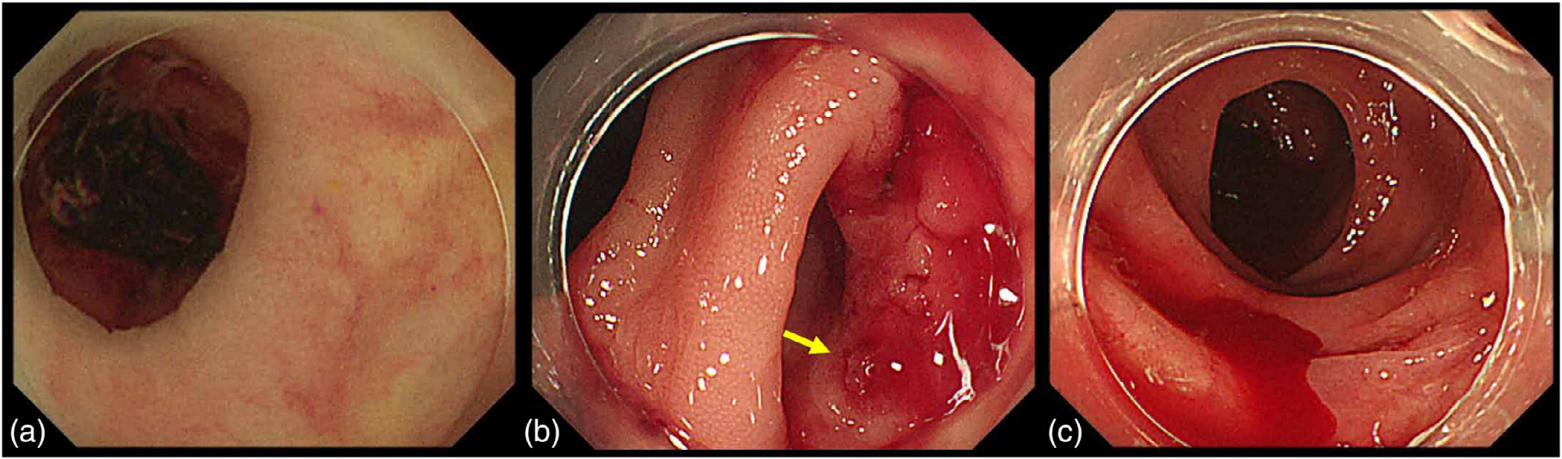
Endoscopic images of stigmata of recent hemorrhage (SRH). (a) Colonic diverticulum with adherent clots. (b) Colonic diverticulum with a visible nonbleeding vessel (yellow arrow). (c) Active bleeding from the colonic diverticulum.

LGIB, including CDB, is characterized by low mortality and high rebleeding rates, with rebleeding being the most critical outcome. Among the eight meta‐analyses, five demonstrated significant identification of the stigmata of recent hemorrhage (SRH) during early colonoscopy (Figure 2); however, none showed a significant reduction in rebleeding (Table 1). Early colonoscopy may improve SRH identification, but its effectiveness depends on the endoscopic hemostasis method employed.^16^, ^17^, ^18^, ^19^, ^27^, ^28^, ^29^, ^30^ Similarly, several large‐scale observational studies focusing specifically on CDB have shown that early colonoscopy does not effectively prevent rebleeding.^8^, ^33^, ^37^

**TABLE 1:** Meta‐analyses evaluating the effectiveness of early colonoscopy for lower gastrointestinal bleeding.

Author	Participants and study design	Rebleeding	Mortality	SRH identification	Endoscopic treatment
Kherad, 2020	Four RCT Thirteen observational studies	NSD OR 1.70 (0.79–3.64)	Early beneficial OR 0.86 (0.75–0.98)	NSD	NSD
Anvari, 2020	Four RCT Nine observational studies	NSD OR 1.59 (0.78–3.21)	Early beneficial RR 0.85 (0.74–0.98)	Early beneficial RR 1.34 (1.05–1.7)	NSD
Tsay, 2020	Four RCT	NSD RR 1.57 (0.74–3.31)	NSD RR 0.93 (0.05–17.21)	NSD RR 1.33 (0.97–1.82)	NSD RR 1.53 (0.67–3.48)
Afshar, 2018	Two RCT Nine observational studies Ten Single‐arm studies	NSD OR 0.89 (0.49–1.62)	NSD OR 0.89 (0.35–2.31)	Early beneficial OR 4.12 (2.00–8.49)	Early beneficial OR 4.17 (2.32–7.49)
Kouanda, 2017	Two RCT Ten observational studies	NSD RR 1.14 (0.74–1.78)	NSD RR 1.17 (0.45–3.02)	NSD RR 1.08 (0.92–1.25)	Early beneficial RR 1.70 (1.08–2.67)
Sengupta, 2017	Two RCT Four observational studies	NSD OR 1.38 (0.85–2.23)	NSD OR 1.64 (0.51–5.32)	Early beneficial OR 2.97 (2.11–4.19)	Early beneficial OR 3.99 (2.59–6.13)
Seth, 2017	Two RCT Four Observational studies	NSD OR 1.18 (0.64–2.16)	NSD OR 0.84 (0.46–1.53)	Early beneficial OR 2.85 (1.90–4.28)	NSD OR 2.60 (0.86–7.84)
Oakland, 2017	One RCT Four observational studies	NSD	NSD	Early beneficial OR 1.86 (1.21–2.86)	Early beneficial OR 3.08 (1.93–4.90)

*Note*: NSD, no significant difference; OR, odds ratio; RR, risk ratio; RCT, randomized controlled trial; SRH, stigmata of recent hemorrhage.

Since 2020, hemostasis techniques with lower rebleeding rates, such as endoscopic band ligation (EBL) and direct clip methods, have been widely adopted, and future advancements in these techniques may further influence the current evidence. While routine early colonoscopy is generally not recommended, it may still be warranted in specific situations such as in patients with shock‐related vital signs or extravasation detected on contrast‐enhanced computed tomography. Notably, subgroup analyses from large multicenter studies on LGIB have reported that patients with a shock index ≥1 or a performance status ≥3 benefit from early endoscopy.^33^ Although these studies targeted LGIB and performance status ≥3 might reflect rectal ulcers, the threshold for early colonoscopy in CDB may need to be lowered for patients with SI ≥1.

### Endoscopic hemostasis for CDB

Among the available treatment options, endoscopic therapy, IVR, and surgery—endoscopic therapy is the least invasive and is therefore widely recommended. This article provides a detailed review of the endoscopic therapies for CDB.

**FIGURE 3:**
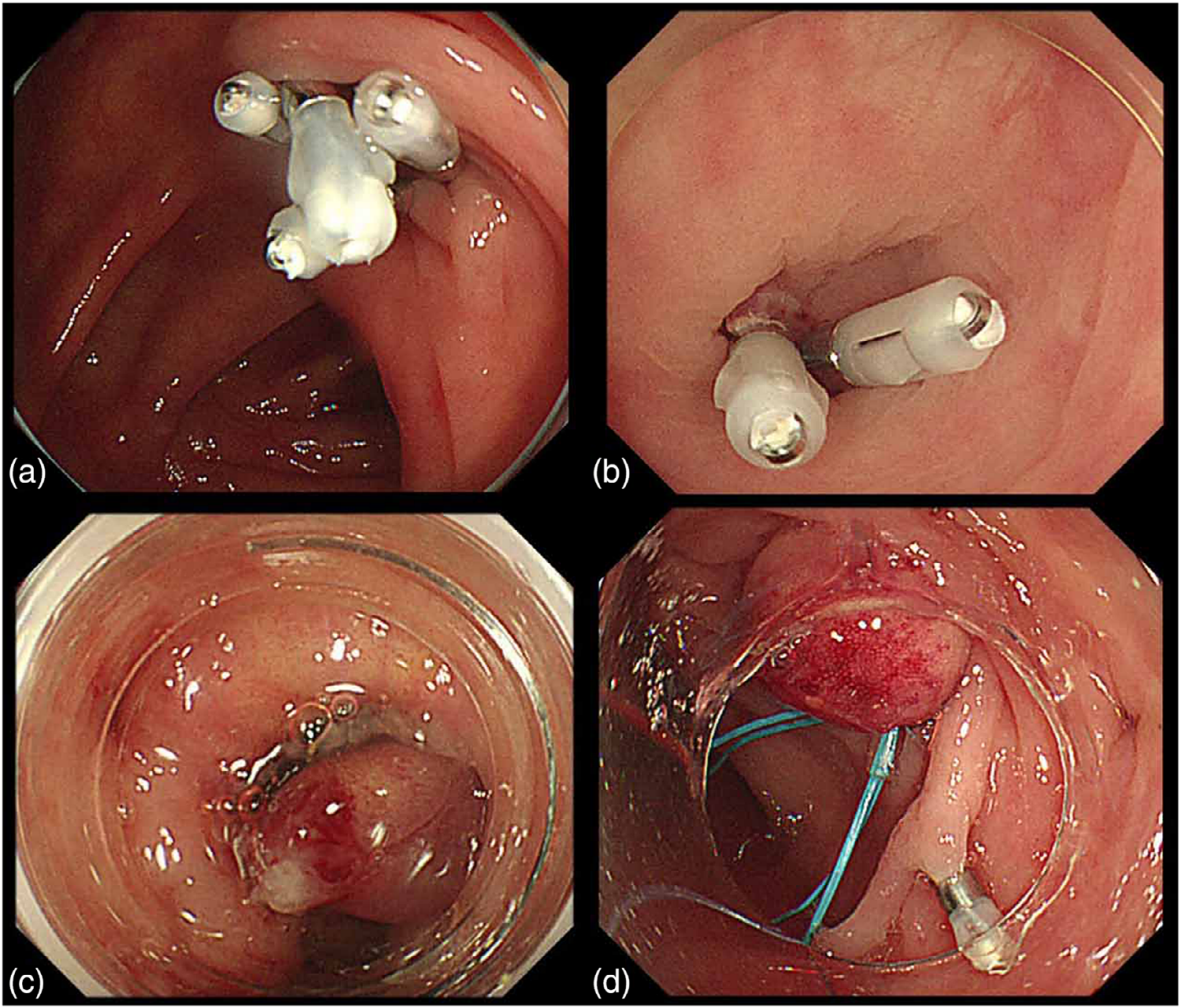
Endoscopic hemostasis for colonic diverticular bleeding (Clipping and ligation methods). (a) The diverticulum is closed in a zipper‐like manner via indirect clip placement (indirect clipping). (b) After direct clip placement of the vessel at the base of the diverticulum (direct clipping). (c) Diverticulum ligated by an elastic O‐ring (endoscopic band ligation). (d) Diverticulum ligated using a detachable snare (endoscopic detachable snare ligation).

Various techniques have been reported for endoscopic hemostasis of CDB, including clipping,^41^, ^42^, ^43^, ^44^, ^45^, ^46^, ^47^, ^48^, ^49^, ^50^, ^51^, ^52^, ^53^, ^54^, ^55^, ^56^, ^57^ ligation methods such as EBL^44^, ^49^, ^51^, ^52^, ^53^, ^54^, ^56^, ^57^, ^58^, ^59^, ^60^, ^61^, ^62^, ^63^, ^64^, ^65^, ^66^ and endoscopic detachable snare ligation (EDSL),^65^, ^66^, ^67^, ^68^, ^69^, ^70^ thermal coagulation,^21^, ^71^, ^72^, ^73^ and epinephrine injection.^21^, ^55^, ^72^, ^73^, ^74^, ^75^ (Figure 3) Recently, novel methods such as Over‐The‐Scope Clip (OTSC; Ovesco Endoscopy, Tübingen, Germany),^76^, ^77^ hemostatic powders^78^, ^79^, ^80^ and self‐assembling peptides (PuraStat; 3‐D Matrix, Tokyo, Japan)^81^, ^82^ have also been introduced for endoscopic hemostasis. Therapeutic outcomes and complications associated with these methods are summarized in Table 2. Numerous studies, including a large multicenter cohort study, have been conducted in Japan to evaluate the efficacy of the clipping and ligation methods. In contrast, evidence regarding other hemostatic techniques remains limited in terms of both the number of reports and cases. Considering the current evidence regarding efficacy and safety, the clipping and ligation methods are prioritized as the primary hemostatic techniques for CDB.

**TABLE 2:** Treatment outcomes of endoscopic hemostasis for colonic diverticular bleeding.

	Endoscopic clipping			Ligation therapy				
	Direct or indirect clipping^41^, ^42^, ^43^, ^44^, ^45^, ^46^, ^47^, ^48^, ^49^, ^50^, ^51^, ^53^, ^54^, ^55^, ^56^	Indirect clipping^52^, ^57^, ^63^, ^69^, ^83^, ^84^	Direct clipping^52^, ^57^, ^63^, ^69^, ^83^	Band ligation^44^, ^49^, ^51^, ^52^, ^53^, ^54^, ^56^, ^58^, ^59^, ^60^, ^61^, ^62^, ^63^, ^64^, ^65^, ^66^, ^94^	Snare ligation^65^, ^66^, ^67^, ^68^, ^69^, ^70^	Thermal coagulation (± epinephrine injection)^21^, ^71^, ^72^, ^73^	Epinephrine injection (± Thermal coagulation ± clipping)^45^, ^55^, ^57^, ^72^, ^73^, ^74^, ^75^	OTSC^76^, ^77^
Number of cases in studies (median; range)	38 (3–1041)	38 (14–681)	34 (14–360)	64 (4–638)	61 (8–283)	10 (3–13)	13 (4–35)	21 (6–36)
Primary hemostasis	88%–100%	94.9%–100%	96.7%–100%	94%–100%	82%–100%	75%–100%	88%–100%	100%
Rebleeding within 30 days	0%–50%	24.2%–35.7%	5.9%–20%	0%–15.5%	4.8%–12.5%	0%–38.5%	0%–38.5%	8.3%–33.3%
Rebleeding within 1 year	18.2%–20.8%	36.4%–39.9%^52^, ^57^, ^83^	32.5%–42.9%	0%–27.8%	29.9%	0%–23%	0%–23.0%	–
Need for interventional radiology	0%–18.8%^44^, ^45^, ^46^, ^47^, ^48^, ^49^, ^51^, ^56^	0%–7.1%^52^, ^57^, ^83^	0%–2.8%^57^	0%–3.2%^52^, ^56^, ^61^, ^94^	0%	0%	0%–8.6%^45^, ^55^	0%
Need for surgery	0%–33.3%	0%–3.6%^52^, ^83^	0%	0%–3.4%^49^, ^56^, ^59^, ^61^, ^94^	0%	0%–30.1%^71^, ^72^	0%–30.1%^45^, ^55^, ^72^, ^74^	0%
Adverse events	Diverticulitis (0.19%)^56^	Diverticulitis (0.6%–0.7%)^89^	0%^89^	Abdominal pain (0%–5.6%)^65^	Abdominal pain (0%–9.5%)^65^, ^68^, ^69^	0%	Perforation (0%–2.9%)^55^	0%
		Sepsis^90^		Diverticulitis (0%–1.6%)^53^, ^56^, ^63^, ^64^, ^94^	Diverticulitis (0%–2.3%)^68^, ^69^, ^70^			
				Perforation (0%–0.93%)^56^, ^66^, ^94^	Perforation^99^			

### Preferred hemostatic methods

#### Endoscopic clipping

**FIGURE 4:**
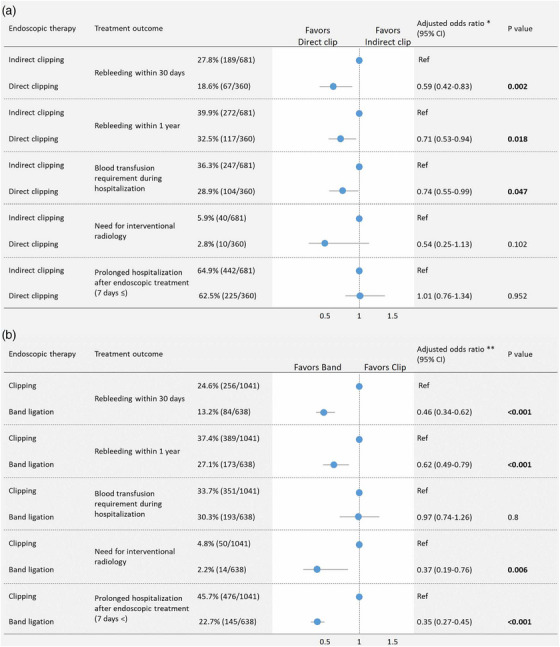
Comparison of treatment outcomes among endoscopic hemostasis techniques in a large‐scale multicenter retrospective cohort study in Japan. (a): Comparison of treatment outcomes between direct clipping and indirect clipping. (b): Comparison of treatment outcomes between endoscopic band ligation and clipping. Note. This figure was based on references 56 and 57. Bold values indicate *p* < 0.05. * Adjusted odds ratios were obtained using multivariate logistic regression analysis. Adjustment for potential confounders included the eight factors of age ≥70 years, sex, heart rate ≥100 bpm, modified Charlson Comorbidity Index ≥2, extravasation on computed tomography, active bleeding, use of distal attachment, and use of water‐jet scope. Most of these were significant in the univariate analysis (*p* < 0.05). ** The multivariate analysis was adjusted for age, sex, and the following 15 factors that were potentially clinically important variables, most of which were found to have at least borderline significance (*p* < 0.10) on univariate analysis: current drinker, systolic blood pressure ≤100 mmHg, loss of consciousness, hemoglobin <12 g/dL, white blood cell >10,000 /µL, blood urea nitrogen >25 mg/dL, antiplatelet use, anticoagulant use, corticosteroid use, extravasation on computed tomography, location, early colonoscopy, bowel preparation, use of distal attachment, and use of water‐jet scope. In the analysis of the need for interventional radiology, multivariate analysis was adjusted for age, sex, and four factors found to be significant (*p* < 0.01) on the univariate analysis between the groups because at least 10 events per confounder were required. CI, confidence interval.

Owing to its simplicity, the clipping technique is widely used for the endoscopic hemostasis of CDB. Clipping methods are classified as: direct clipping which involves grasping the exposed vessel and indirect clipping, which closes the diverticular orifice.^52^, ^57^, ^63^, ^69^, ^83^, ^84^ A large multicenter cohort study demonstrated that direct clipping significantly reduced the risk of early rebleeding (< 30 days) compared with indirect clipping (18.6% vs. 27.8%, adjusted odds ratio [AOR] 0.592, *p* = 0.002; Figure 4A).^52^, ^57^ However, subgroup analyses revealed that rebleeding rates did not significantly differ between direct and indirect clipping groups in cases with active bleeding or left‐sided colon.^57^ Effective direct clipping requires precise grasping of the bleeding point at the base of the diverticulum, which is technically challenging.^52^, ^57^ To address this, methods combining a distal attachment cap and underwater observation,^52^, ^85^, ^86^ as well as the use of endoclips with a reopening function,^87^, ^88^ have been reported. Endoscopic images and video depictions of direct clipping with these devices for CDB are available in a recently published series.^52^, ^85^, ^86^, ^87^, ^88^ Complications of clipping include diverticulitis, reported at a rate of 0.19% (2/1041) across all clipping methods (direct and indirect).^56^ The complication rates (diverticulitis) for direct and indirect clipping are 0.3% (2/673), and 0% (0/353), respectively.^89^ Additionally, there has been a case report of sepsis following the indirect clipping for CDB.^90^

#### Ligation therapy

CDB can originate from two distinct locations within the diverticulum: the neck or base.^91^, ^92^ EBL is a ligation technique approved by the Japanese insurance for CDB and enables mechanical hemostasis regardless of the location of the bleeding point (neck or base). A large multicenter cohort study showed that EBL was independently associated with a reduced risk of early rebleeding (13.2% vs. 24.6%, AOR 0.46, *p* < 0.001) and late rebleeding (27.1% vs. 37.4%, AOR 0.62, *p* < 0.001) compared with clipping including direct and indirect (Figure 4B).^56^ Moreover, EBL showed significantly lower rebleeding rates regardless of the presence of active bleeding.^56^ Similarly, other studies have reported that EBL has significantly lower rates of 30‐day rebleeding,^44^, ^53^, ^54^, ^93^ 1‐year rebleeding,^49^, ^51^, ^63^, ^93^ and transition to IVR or surgery (Table 2).^56^, ^93^ However, EBL requires reinsertion of the endoscope to attach the ligation device, leading to significantly longer procedure times than clipping.^52^, ^94^

Another ligation technique, EDSL, allows immediate hemostasis at the identified bleeding point and is simpler to perform than EBL. Similar to EBL, EDSL has been reported to achieve significantly lower 30‐day rebleeding rates than clipping (6.8% vs. 23.0%; *p* = 0.031).^69^ However, EDSL for the hemostasis of CDB is not approved by Japanese insurance and requires Ethics Committee approval for specific clinical research and sufficient informed consent from the patient.

Complications associated with ligation therapy include diverticulitis and delayed perforation, both of which have been reported in patients undergoing EBL and EDSL (Table 2). Diverticulitis has been reported to resolve with conservative treatment.^56^, ^68^, ^69^, ^70^, ^95^ In contrast, five cases of delayed perforation have been reported in Japan, all requiring surgical intervention.^94^, ^96^, ^97^, ^98^, ^99^ These cases were characterized by the involvement of the left‐sided colon (sigmoid colon, two cases; descending colon, three cases), long‐term steroid use for collagen disease in two cases, and maintenance dialysis in an elderly patient in another. The risk of delayed perforation should be carefully considered when performing ligation therapy in patients with impaired wound healing or left‐sided colonic lesions. A large multicenter cohort study in Japan reported a low incidence rate of complications after EBL, with perforation occurring in 0.31% (2/638) and diverticulitis in 0.16% (1/638) of cases. These rates did not differ significantly from those of clipping (perforation: 0% [0/1041], diverticulitis: 0.19% [2/1041]; Table 2).^56^ However, bowel perforation remains a serious complication requiring surgical intervention, and it is important to balance efficacy and safety when choosing a treatment.

### Comparison of clipping and ligation therapy outcomes

**FIGURE 5:**
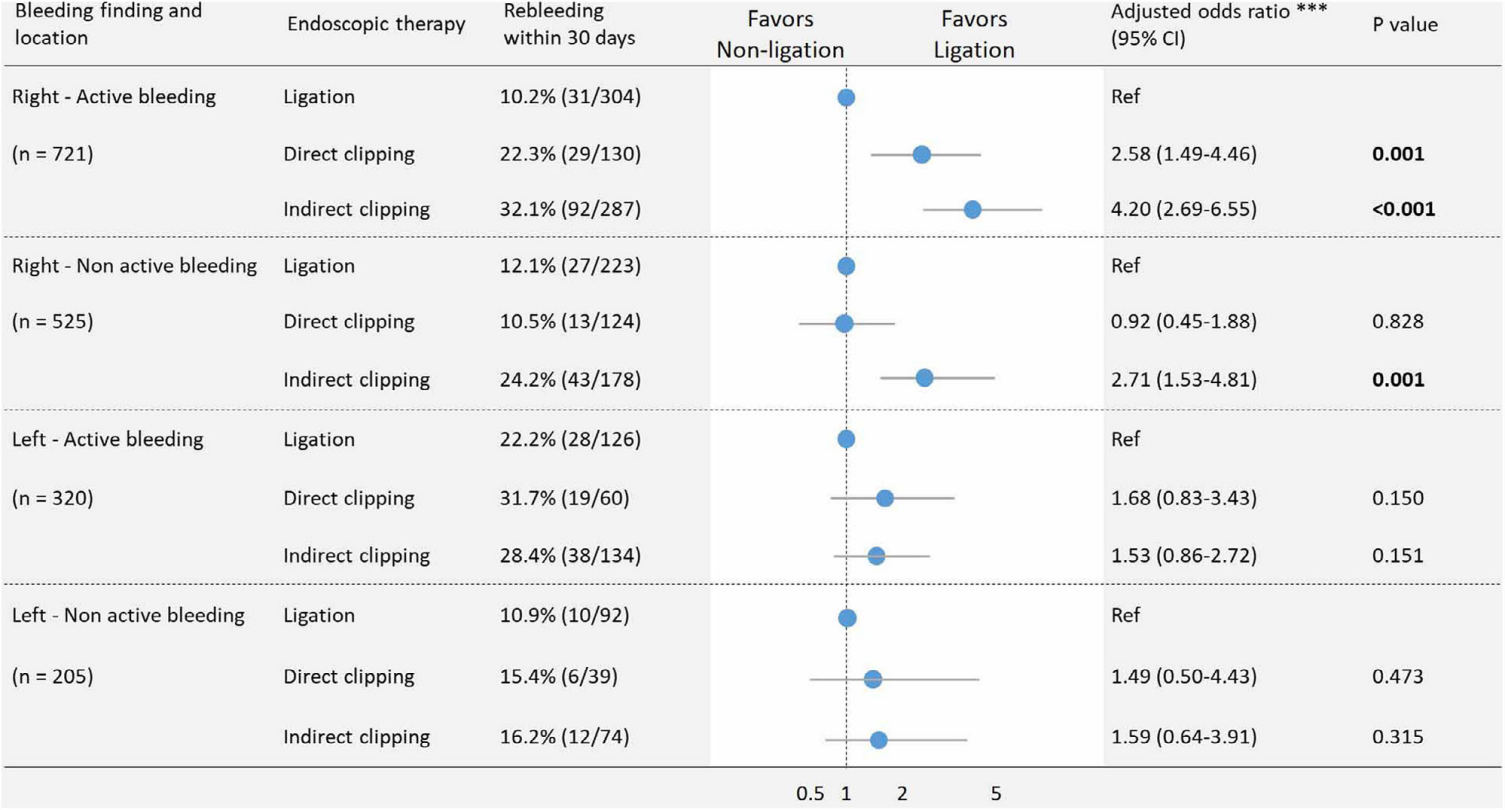
Differences in early rebleeding rates by bleeding findings and locations in a large‐scale multicenter retrospective cohort study of colonic diverticular bleeding in Japan. Note. This figure was created based on reference 88. Ligations included the snare and band ligation methods. Bold values indicate *p* < 0.05. ***Twelve variables that had the potential to be clinically important variables were integrated as covariates by calculating the propensity score, including performance status, systolic blood pressure ≤100 mmHg at admission, pulse rate ≥100 bpm at admission, loss of consciousness, antiplatelet use, anticoagulant use, corticosteroid use, history of colonic diverticular bleeding, early colonoscopy, bowel preparation, use of a distal attachment, and use of a waterjet scope. Thus, the multivariate analysis was adjusted for age, sex, and propensity score‐based covariates. Exact logistic regression analysis.

Although no randomized controlled trials have compared the efficacy of clipping and ligation therapy, recent meta‐analyses^93^, ^100^ and large multicenter cohort studies from Japan have provided valuable insights. The key findings are: (1). EBL achieved lower rebleeding rates than clipping (13.2% vs. 24.6%, AOR 0.46, *p* < 0.001). ^56^, ^93^ (2). Direct clipping was associated with lower rebleeding rates than indirect clipping (18.6% vs. 27.8%; AOR, 0.592; *p* = 0.002),^57^ (3). No significant difference in the rebleeding rate was observed between EBL and EDSL (11.3% vs. 11.7%, OR 1.04, *p* = 0.93).^66^ (4). In cases of active bleeding in the right colon, the rebleeding rate was significantly lower with ligation therapy, direct clipping, and indirect clipping (10.2% vs. 22.3% vs. 32.1%; Figure 5).^89^ (5). For non‐active bleeding in the right colon, no significant difference in rebleeding rates existed between ligation therapy and direct clipping (12.1% vs. 10.5%; Figure 5).^89^ (6). In the left‐sided colon, regardless of the presence of active bleeding, no significant differences in rebleeding rates were observed among ligation therapy, direct clipping, and indirect clipping (Figure 5).^89^ These findings suggest that the choice of treatment should be based on factors such as the location of the bleeding point and the presence of active bleeding.

### Treatment selection strategy between clipping and band ligation

**FIGURE 6:**
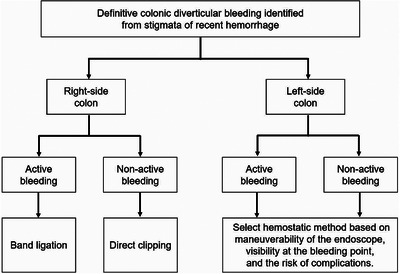
Endoscopic hemostatic methods for colonic diverticular bleeding considered based on the bleeding findings and location.

Rebleeding rates following endoscopic treatment for CDB have been shown to vary depending not only on the treatment method but also on the bleeding findings and location.^56^, ^57^, ^89^ Therefore, it is crucial to consider these factors when selecting the treatment approach. Based on these data, we developed an algorithm to guide treatment selection based on the bleeding site (right or left colon; Figure 6).

These findings suggest that, in the right colon, the strategy of direct clipping when clip placement at the bleeding point is feasible and EBL when direct clipping is not feasible because of a narrow diverticular orifice or active bleeding is reasonable in terms of effectiveness and efficiency. In contrast, these findings suggest that either hemostatic method is acceptable for the left‐sided colon.^56^, ^57^, ^89^ Recently, a single‐center retrospective study evaluated the efficacy of direct clipping when clip placement at the bleeding point was feasible, and EBL when direct clipping was not feasible. There was no significant difference in the rebleeding rates between clipping and EBL (15.5% vs. 13.0%, *p* = 0.619).^94^ This supports the findings of previous studies.^56^, ^57^, ^89^

### Other treatment methods

#### Over‐the‐scope clip

The OTSC achieved hemostasis by grasping the diverticulum suctioned into the distal cap using a shape memory clip. A unique feature of OTSC is that it preserves blood flow within the grasped diverticulum, making it less prone to necrotic changes.^77^ Additionally, due to its ability to “jump” forward by approximately 4 mm, OTSC has been reported to be effective even in cases where adequate suction of the diverticulum is difficult, such as after rebleeding following EBL.^101^, ^102^ However, the available data on treatment outcomes as initial therapy are limited to two studies with small sample sizes, reporting 30‐day rebleeding rates ranging from 8.3% to 33.3% (Table 2). A case series has also been reported in which OTSCs were used as a rescue therapy for CDB that recurred after endoscopic hemostasis with either clipping or EBL.^103^ In this series, immediate hemostasis was successfully achieved in all four cases. However, three patients subsequently experienced rebleeding during hospitalization, requiring IVR or surgical intervention. OTSCs also have economic limitations owing to their high costs. The price of an OTSC device is ¥79,800 (excluding tax; approximately 532 USD, based on an exchange rate of 150 JPY/USD) compared to ¥10,000 (excluding tax; approximately 67 USD) for an EBL and ¥1000–2000 (excluding tax; approximately 7–13 USD) per clip, depending on the type. Indications regarding OTSC should be carefully considered in clinical practice, given the limited evidence supporting OTSC and its high cost.

#### Epinephrine injection

Epinephrine injection involves endoscopic hemostasis with 1 or 2 mL per injection of diluted epinephrine (1:10,000 or 1:20,000 mixed with saline) into the neck of the bleeding diverticulum. The effect of epinephrine injection alone is pharmacologically temporary, and long‐term hemostasis is not expected. Therefore, it is reported in combination with clipping or thermal coagulation methods (Table 2).^45^, ^55^, ^72^, ^73^, ^75^ The studies comparing a group treated with clipping alone and a group treated with a combination of clipping and epinephrine injection found no significant difference in early rebleeding rates (8.3% vs. 18.8%, *p* = 0.653).^55^, ^57^ However, delayed perforation was observed in the combination group (2.9%, 1/35; Table 2).^55^ From the perspectives of efficacy and safety, epinephrine injection is not recommended.

#### Thermal coagulation

Multiple thermal coagulation modalities are available, including monopolar/bipolar hemostatic forceps, bipolar probes, and heater probes. As monopolar electrocoagulation causes extensive thermal damage and carries the risk of perforation when performed in a false diverticulum of the colon, bipolar probes are generally used for CDB. Bipolar probes have been used in the United States and other countries but are not available in Japan. Thermal coagulation has rarely been reported in Japan. Reports often describe its use in combination with epinephrine injections.^21^, ^71^, ^72^, ^73^ Data on treatment outcomes are limited to studies from the United States, with small sample sizes of approximately 10 cases. The reported 30‐day rebleeding rates ranged from 0% to 38.5%, and the surgical transition rates ranged from 0–30.1% (Table 2). This procedure is not recommended owing to the risk of perforation when thermal coagulation is performed on a colonic diverticulum lacking a muscular layer.^104^

#### Self‐assembling peptide

A novel synthetic self‐assembling peptide, PuraStat (3‐D Matrix Ltd.), was introduced as a surgical or endoscopic hemostatic agent. ^105^, ^106^ The peptide self‐assembles into an extracellular scaffold matrix when activated by a pH change associated with exposure to blood. The matrix adheres to and seals the blood vessels, thereby achieving hemostasis as a mechanical barrier. Additionally, the activated matrix promotes tissue proliferation and facilitates effective mucosal healing; however.^106^ Evidence for the use of PuraStat in CDB remains limited. Case reports have documented its effectiveness in achieving hemostasis.^81^, ^107^, ^108^ A recent multicenter pilot study involving 25 patients with CDB reported a 100% success rate for endoscopic hemostasis using PuraStat, either as monotherapy or in combination with other methods such as EBL or clipping. Notably, the 30‐day rebleeding rate was significantly lower in patients treated with PuraStat than in those treated without it (4.0% vs. 20.9%, *p* = 0.047), suggesting its potential to reduce the risk of rebleeding.^109^

## SRH IDENTIFICATION TECHNIQUES

Identifying the SRH in CDB is crucial, as it contributes to reducing the rebleeding rate.^11^, ^89^ Although significant advancements have been made in hemostatic techniques for CDB in recent years, the SRH identification rate during initial colonoscopy remains low at 30.9%,^1^ as reported in a large multicenter cohort study in Japan. This remains a challenge in the management of CDB. Studies investigating factors associated with SRH identification have shown that performing colonoscopy within 24 h,^21^, ^22^, ^33^, ^110^ an endoscopist with experience in more than 1,000 colonoscopies,^110^ using a distal attachment cap,^60^, ^110^ using a water‐jet scope,^110^ utilizing a nontraumatic tube,^111^ and conducting endoscopic observation for more than 19 min^112^ are significantly associated with increased SRH identification rates. Furthermore, a multicenter nationwide study in Japan identified seven predictors of SRH: three colonoscopic factors (early colonoscopy [<12 h], use of an attachment cap, and use of a water‐jet device), absence of abdominal pain, elevated PT‐INR ≥2.0, extravasation on computed tomography, and use of direct oral anticoagulants.^113^ Therefore, in addition to improving endoscopic techniques, clinical factors may also serve as important indicators. As an innovation in endoscopic techniques, the use of a long hood (MAJ‐663; Olympus) has been reported to be effective for diagnosing CDB compared to a short hood.^114^ Additionally, water immersion observation^86^, ^115^ and gel immersion endoscopy^116^, ^117^ have also been reported as case reports of techniques for improving SRH identification. Bowel preparation with polyethylene glycol enhances cecal intubation rates while ensuring safety, making it desirable for CDB colonoscopy.^118^ On the other hand, although colonoscopy within 24 h contributes to SRH identification, there is currently no data demonstrating an improvement in the critical outcome of rebleeding (Table 1). Therefore, at present, early colonoscopy cannot be actively recommended.

## MANAGEMENT OF ANTITHROMBOTIC MEDICATIONS

### Antiplatelet Agents

There is limited evidence specifically regarding antithrombotic medication management in CDB. Therefore, recommendations are extrapolated from studies on LGIB. Two major retrospective cohort studies have investigated the effects of antiplatelet agent discontinuation in LGIB. A study of 295 patients with LGIB who were taking aspirin for secondary prevention of thromboembolic disease demonstrated that while aspirin continuation increased the risk of rebleeding compared to discontinuation, it resulted in fewer serious cardiovascular events and deaths.^119^ Additionally, a study of 2528 LGIB patients on antiplatelet therapy found that discontinuation of antiplatelet agents did not reduce the risk of rebleeding.^120^ Based on these findings, it is suggested that antiplatelet agents should not be discontinued in patients with LGIB who are taking these medications for secondary prevention of thromboembolic disease. However, temporary discontinuation may be considered in cases of persistent bleeding or recurrent episodes of bleeding, with prompt reinitiation following confirmed hemostasis. For patients taking aspirin for primary prevention of thromboembolic disease, discontinuation may be considered following LGIB.^104^, ^121^

### Dual antiplatelet therapy

No studies have compared outcomes between continuing DAPT, discontinuing it, or switching from DAPT to a single agent in patients with LGIB on DAPT. Although not specific to gastrointestinal bleeding, 75% of patients who discontinued DAPT after coronary stent placement developed thromboembolism within 10 days, whereas only 6% of those who discontinued only thienopyridines experienced thromboembolism.^122^ Based on these findings, it is suggested that LGIB patients on DAPT continue antiplatelet therapy with aspirin alone while discontinuing other antiplatelet agents for 5–7 days.^104^ However, discontinuing DAPT within one month after stent placement for acute coronary syndrome is not recommended.^123^, ^124^

### Anticoagulants

There are no studies evaluating the safety of anticoagulant temporary discontinuation after the onset of LGIB. As discontinuation of anticoagulants is associated with thromboembolic events and may cause irreversible complications, permanent discontinuation should be avoided and continuation of anticoagulant therapy should be considered even after LGIB. However, temporary discontinuation may be considered in cases of persistent bleeding or recurrent episodes of bleeding. In such cases, patients and their families should be informed about the risk of thromboembolic events, with prompt reinitiation following confirmed hemostasis.

### Combination of multiple antithrombotic medications (excluding DAPT)

There is no evidence regarding the discontinuation of each drug when multiple antithrombotic medications are used. Decisions should be made after consulting with and closely collaborating with various specialists (hematology, cardiology, neurology, and gastroenterology).

## CONCLUSIONS

Since 2020, multiple large‐scale studies have significantly expanded our understanding of CDB. Epidemiological data revealed that the number of patients with CBD is increasing owing to an aging population, highlighting the importance of establishing the best management options for CDB in Japanese gastrointestinal practice. Meanwhile, increasing evidence supports endoscopic treatments such as precisely applied direct clipping and EBL, which can maintain rebleeding rates of approximately below 15%. Despite these advances, the most pressing challenge remains improving the detection rate of SRH. It is important to address these new findings in the future to develop more efficient techniques and diagnostic algorithms to identify bleeding sources.

## CONFLICT OF INTEREST STATEMENT

None.

## Supporting information



ICMJE Disclosure form
